# Supplementary material for: Prediction of Incomplete Response of Primary Tumour Based on Clinical and Radiomics Features in Inoperable Head and Neck Cancers after Definitive Treatment
Source: J Pers Med. 2022 Jun 30;12(7):1092. doi: 10.3390/jpm12071092 (PMC9317569; doi:10.3390/jpm12071092)
Supplement: Supplementary file 1 [file jpm-12-01092-s001.zip › Supplementary files /Ethic Committee decision.pdf]

POZNAN UNIVERSITY OF MEDICAL SCIENCES

BIOETHICS COMMITTEE

no. 70, Bukowska Str.
60-812, Poznań
Poland

Phone: + 48 61 854 73 36
e-mail: bioetyka.ump@ump.edu.pl
www. bioetyka.ump.edu.pl

Poznań, May 6, 2022

KB – 367/22

CONFIRMATION

I herby confirm that scientific research entitled:

„Prediction of incomplete response of primary tumour based on clinical and radiomics features in inoperable head and neck cancers after definite treatment".

conducted by **Joanna Kaźmierska**

is not a medical experiment.

According to the Polish law and GCP regulations
this research does not require approval of the Bioethics Committee
at Poznan University of Medical Sciences.

M. Krawczyński

Chairman of the Committee
Professor Maciej Krawczyński, MD, PhD
